# Isomorphism of Sr[Li_3_AlO_4_] and Sr[Li_3_GaO_4_] – Syntheses, Crystal Structure, and Europium(II) Luminescence

**DOI:** 10.1021/acs.chemmater.4c01382

**Published:** 2024-07-18

**Authors:** Johannes G. Volpini, Mark Vorsthove, Christiane Stoll, Daniel Bichler, Markus Seibald, Hubert Huppertz

**Affiliations:** † Department of General, Inorganic and Theoretical Chemistry, University of Innsbruck, Innrain 80-82, Innsbruck AT-6020, Austria; ‡ ams-OSRAM International GmbH, Mittelstetter Weg 2, Schwabmünchen D-86830, Germany

## Abstract

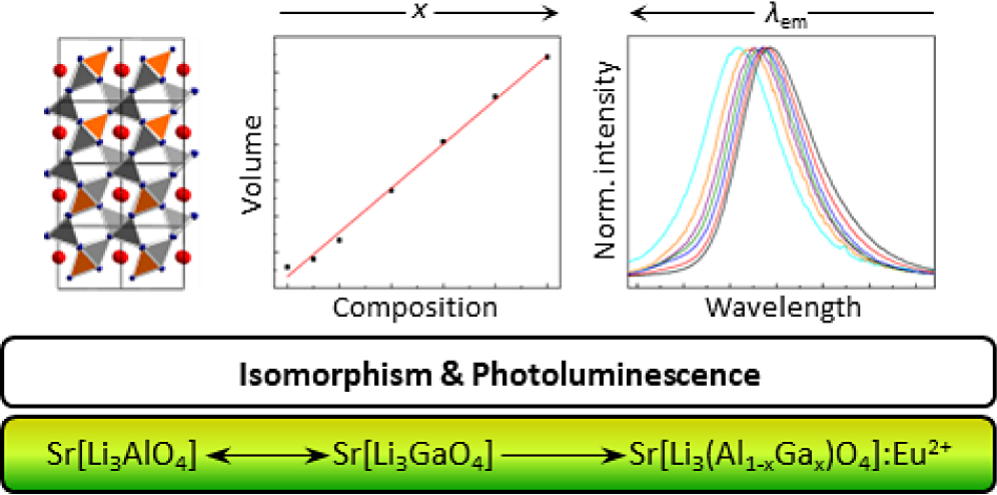

While highly efficient red-emitting inorganic phosphors have been discovered in the substance class of alkaline earth oxo(nitrido)lithoaluminates, new narrow-band green- and yellow-emitting components are being sought to improve the performance of phosphor-converted light-emitting diodes (pc-LEDs). Various solid-state reactions were carried out under protective gas atmosphere in nickel crucibles and sealed tantalum ampules to synthesize Sr[Li_3_AlO_4_], Sr[Li_3_GaO_4_], and five substitutional derivates of Sr[Li_3_(Al_1–*x*_Ga_*x*_)O_4_] at moderate temperatures. The observation of a linear increase in the unit cell parameters as a function of the increasing gallium mole fraction *x* in Sr[Li_3_(Al_1–*x*_Ga_*x*_)O_4_] revealed Vegard behavior in the solid-solution series, which was derived from powder X-ray diffraction data. The isomorphic crystallization of the new oxolithogallate Sr[Li_3_GaO_4_] and the known oxolithoaluminate Sr[Li_3_AlO_4_] in an ordered variant of the U[Cr_4_C_4_] aristotype was verified on the basis of powder and single-crystal X-ray diffraction data. Photoluminescence spectroscopy was used to investigate the narrow-band emissions in the substitution series of Eu^2+^-activated Sr[Li_3_(Al_1–*x*_Ga_*x*_)O_4_] under blue-light excitation. The emission maximum was shifted to higher energies as the gallium mole fraction increased. Peak wavelengths were observed at λ_em_ = 572 nm (fwhm equals 47 nm, 1446 cm^–1^, 0.18 eV) for yellow-emitting Sr[Li_3_AlO_4_]:Eu^2+^ and at λ_em_ = 554 nm (fwhm equals 49 nm, 1589 cm^–1^, 0.20 eV) for green-emitting Sr[Li_3_GaO_4_]:Eu^2+^. Sr[Li_3_AlO_4_]:Eu^2+^ has excellent thermal quenching resistance with a photoluminescence emission intensity of >93% at *T* = 423 K relative to the room temperature value, making this inorganic phosphor a potential candidate for solid-state lighting applications.

## 1 Introduction

Over the past decade, the substance class of alkaline earth oxo(nitrido)lithoaluminates has increasingly attracted the interest of researchers in the field of inorganic phosphors, when Eu^2+^-activated Ca[LiAl_3_N_4_],^1^ Sr[LiAl_3_N_4_],^2^ Ca_18.75_Li_10.5_[Al_39_N_55_],^3^ Sr_4_[LiAl_11_N_14_],^4^ and Sr[Li_2_Al_2_O_2_N_2_]^5^ were discovered. The interconfigurational transitions from the excited 4f^6^5d^1^ states to the 4f^7^ ground state of the Eu^2+^ activator ion in the above-mentioned compounds lead to narrow-band emissions in the red spectral region of the visible spectrum, making some of them promising candidates for applications in phosphor-converted light-emitting diodes (pc-LEDs).^6^ In the cases of Ca[LiAl_3_N_4_], Sr[LiAl_3_N_4_] and Sr[Li_2_Al_2_O_2_N_2_], detailed spectroscopic studies have been carried out to explore the coordination environments of Eu^2+^.^7−11^ Except for Ca_18.75_Li_10.5_[Al_39_N_55_] and Sr_4_[LiAl_11_N_14_], the crystal structures of the described oxo(nitrido)lithoaluminates are ordered variants of the U[Cr_4_C_4_] aristotype.^12^ The rediscovery of the U[Cr_4_C_4_]-type structure or closely related structures was driven by the observation of (ultra-) narrow-band emissions emanating from Eu^2+^-activated alkaline-rich lithosilicates^13−17^ and alkaline earth-rich lithoaluminates.^1,2,5^ The nitridolithoaluminate Sr[LiAl_3_N_4_] crystallizes in the ordered variant, which was first described for the isotypic crystal structure of Cs[Na_3_PbO_4_].^18^ The complete replacement of nitrogen by oxygen and the partial exchange of aluminum by lithium in Sr[LiAl_3_N_4_] leads to Sr[Li_3_AlO_4_]. The nitridolithoaluminate and the oxolithoaluminate exhibit isotypic crystal structures,^19−22^ which differ not only in the anionic ligands but also in the distribution of the tetrahedrally coordinated cations in the highly condensed rigid framework.^2,22^ A detailed description of the solid-state synthesis, single-crystal X-ray diffraction data and the luminescence properties of Eu^2+^-activated Sr[Li_3_AlO_4_] is presented in the literature,^22^ but no database-accessible X-ray diffraction data have been published to date. The first experimental single-crystal X-ray diffraction data of crystals with chemical compositions close to that of Sr[Li_3_AlO_4_] were presented by A. Ooishi and colleagues.^23^ They demonstrated on different crystallites that disordered average structures can be observed in Sr_*x*_[Li_2+*x*_Al_2–*x*_O_4_], which are closely related to the U[Cr_4_C_4_]-type structure, but require the description of commensurate and incommensurate modulated superstructures. Once a phosphor material has been structurally elucidated and its luminescence properties are known, tuning the emission in the meaning of changing the spectral position or spectral bandwidth is a common way to improve its luminescence performance for future applications, or, conversely, to prove its unsuitability for these. Frequently used strategies to achieve the described objectives are based on the selection of the activator, the variation of its concentration, and the complete replacement or partial substitution of the cation(s) or anion(s) in the host structure.^24^ As already shown in the introduction, numerous representatives of quaternary alkaline earth oxo(nitrido)lithoaluminates have been discovered and structurally elucidated. Also, several crystallographic studies have been performed in the corresponding quaternary lithogallates^25,26^ and in the field of nitridogallates, e.g., (Ca,Sr)_3_Ga_2_N_4_^27^ and (Sr,Ba)_3_Ga_3_N_5_^27,28^ presented by the groups of F. J. DiSalvo and Clarke and W. Schnick et al., respectively. Based on solid-state synthesis at moderate temperature, aluminum was completely replaced by gallium in Sr[Li_3_AlO_4_], which yields the hitherto unknown quaternary oxolithogallate Sr[Li_3_GaO_4_]. Powder X-ray diffraction experiments in the cationic substitution series of Sr[Li_3_(Al_1–*x*_Ga_*x*_)O_4_] demonstrate that a solid-solution series is present, and therefore the crystal structures of Sr[Li_3_AlO_4_] and Sr[Li_3_GaO_4_] can be described as isomorphous in the sense of the definition given by P. H. Groth.^29^ The claim of isomorphism is supported by the observation of Vegard behavior^30,31^ and the emission tunability in the substitution series of Eu^2+^-activated Sr[Li_3_(Al_1–*x*_Ga_*x*_)O_4_]. Finally, the thermal quenching behaviors of the aluminate and gallate representatives were investigated to evaluate their use as inorganic phosphors in solid-state lighting (SSL) applications.

## 2 Experimental Section

### 2.1 Synthesis

**Figure 1:**
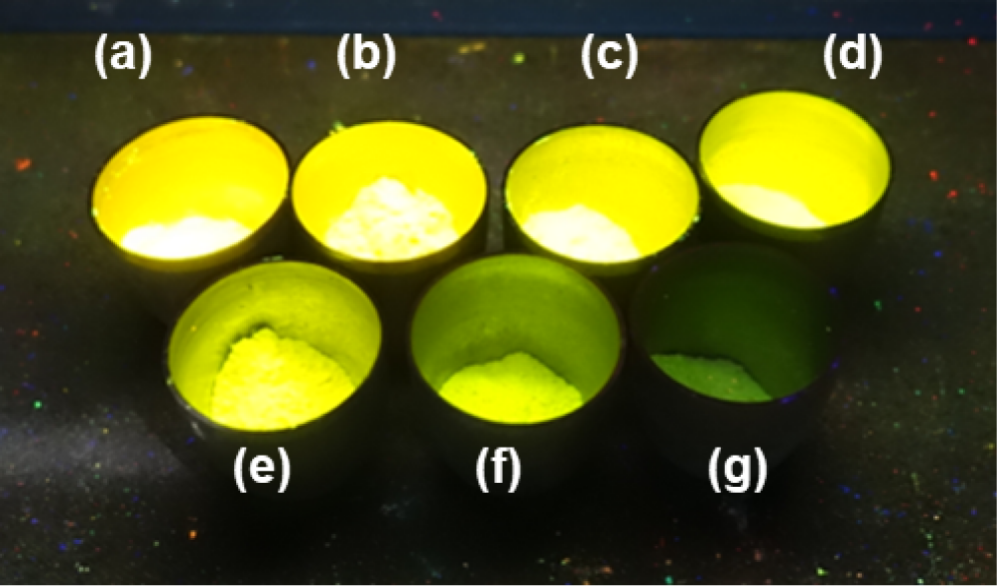
Eu^2+^-activated powder samples of (a) Sr[Li_3_AlO_4_], (g) Sr[Li_3_GaO_4_], and the substitutional derivates of Sr[Li_3_(Al_1–*x*_Ga_*x*_)O_4_] with the nominal gallium mole fraction *x* being equal to (b) 0.1, (c) 0.2, (d) 0.4, (e) 0.6, and (f) 0.8 in nickel crucibles irradiated with UV light (λ ≈ 254 nm).

The starting materials SrO, Li_2_O, and SrAl_2_O_4_ were prepared in corundum crucibles in a chamber furnace (Nabertherm, Lilienthal, Germany). SrCO_3_ (99.6%, SL 300, Solvay) and Li_2_O_2_ (97.5%, Albemarle) were fired in a nitrogen atmosphere (Linde Gas, Stadl-Paura, Austria) for 8 h at 1573 and 673 K, respectively. Equimolar amounts of SrCO_3_ and Al_2_O_3_ (≥99.8%, Evonik) were heated in a forming gas atmosphere (nitrogen/hydrogen, 92.5/7.5%, Linde Gas, Stadl-Paura, Austria) to 1473 K with 1.7 K·min^–1^ and the temperature was kept for 6 h. The reaction between Sr_2_N (1.5 mol, 99.5%, Materion) and AlN (2 mol, 99%, Tokuyama) was used to synthesize Sr_3_Al_2_N_4_ in a flow tube furnace (Carbolite Gero, Neuhausen, Baden-Württemberg, Germany) by firing the mixture in a nitrogen flow in a corundum crucible for 6 h at 1373 K. Single crystals and powders of Sr[Li_3_AlO_4_] were synthesized from SrO, Sr_3_Al_2_N_4_, SrAl_2_O_4_, and Li_2_O. Powder samples of Sr[Li_3_(Al_1–*x*_Ga_*x*_)O_4_] with the nominal gallium mole fraction *x* being equal to 0.1, 0.2, 0.4, 0.6, 0.8, and 1 were prepared according to the previous approach using Ga_2_O_3_ (>99.9%, Vollmer) or GaN (99.99%, abcr) as the gallium source. The stoichiometric ratios of the starting materials used are listed in Table S1. Li_2_B_4_O_7_ (0.6 g, 0.0035 mol) was added to each reaction mixture as a mineralizing agent and additional source of lithium. The starting materials were mixed in an agate mortar in a glovebox filled with inert gas (H_2_O <1 ppm, O_2_ <1 ppm, MBraun, Garching, Germany) and crushed in closed plastic containers for 12 h using ZrO_2_ grinding bowls. The samples were transferred into nickel crucibles, which were closed with lids and placed in a chamber furnace. After heating the samples in a forming gas atmosphere to 1073 K with 4.2 K·min^–1^, the temperature was kept for 4 h, and then cooled to room temperature by turning off the furnace. Single crystals of Sr[Li_3_GaO_4_] were obtained from the reaction between SrO (4.15 g, 0.04 mol), Li_2_CO_3_ (1.48 g, 0.02 mol, ≥99.0%, Merck), Ga_2_O_3_ (3.75 g, 0.02 mol, 99.998%, Strem Chemicals), and lithium metal (1.11 g, 0.16 mol, 99%, Merck). The starting materials for the oxolithogallate were mixed in an agate mortar and filled into tantalum ampules. The tantalum ampules were sealed in an inert gas atmosphere (Linde Gas, Stadl-Paura, Austria) using a tungsten welding system (Fronius International, Pettenbach, Austria) and transferred into a silica tube filled with 400 mbar argon. The silica tube was placed in a self-assembled horizontal tube furnace (Controller 3216, Eurotherm, Durrington, United Kingdom), heated to 1123 K with 3.0 K·min^–1^, and the temperature was kept for 8 h. After cooling the sample to 973 K with 0.1 K·min^–1^, the tube furnace was turned off. The Eu^2+^-activated compounds were prepared using Eu_2_O_3_ (≥99.99%, Strem Chemicals) as an activating agent at nominal concentrations of approximately 0.5 mol % (Sr formally substituted by Eu). The reduction of Eu^3+^ to Eu^2+^ was most likely achieved by the reducing forming gas atmosphere in the nickel crucibles and the lithium metal in the sealed tantalum ampules, respectively. Crystals and powders of the products appeared slightly green to yellow in daylight, showing luminescence with a green-to-yellow color impression when irradiated by UV to blue light. Figure 1 shows the powder products in nickel crucibles that were irradiated with a UV lamp (λ ≈ 254 nm, CAMAG, Muttenz, Switzerland). The powder products were handled and stored under atmospheric conditions or, in the case of single crystals, in perfluorinated oil. Powder X-ray diffraction measurements have confirmed the presence of Sr[Li_3_(Al_1–*x*_Ga_*x*_)O_4_] even after months of storage under ambient conditions.

### 2.2 Single-Crystal X-Ray Diffraction

Single crystals of Sr[Li_3_AlO_4_] and Sr[Li_3_GaO_4_] were selected under a stereomicroscope (Leica Microsystems, Wetzlar, Germany) and the diffraction data of suitable individuals were collected on a D8 Quest Kappa X-ray diffractometer (Bruker, Billerica, USA). The X-ray source used was a microfocus X-ray tube (Incoatec, Geesthacht, Germany) with a copper anode (Cu K-L_2,3_, λ = 1.54178 Å) and the diffracted X-ray photons were recorded with a Photon II detector (Bruker, Billerica, USA). To elucidate the crystal structure of Sr[Li_3_AlO_4_], the unit cell refinement was performed with SAINT (v. 8.38A)^32^ and SADABS (v. 2016/2)^33^ was used for the multiscan absorption correction, both implemented in APEX (v. 2.0).^34^ In the case of Sr[Li_3_GaO_4_], the program APEX (v. 3.0)^35^ was used for data collection and processing including the unit cell refinement with SAINT (v. 8.40A)^36^ and the multiscan absorption correction using SADABS (v. 2016/2).^33^ OLEX (v. 2–1.5)^37^ was used to solve the crystal structures in the triclinic space group *P*1̅ (no. 2) with SHELXT (v. 2014/5).^38^ The structure models were refined using full-matrix least-squares against *F*^2^ in SHELXL (v. 2018/3).^39^ PLATON (v. 230318)^40^ routines were used to check the determined space group with ADDSYM and STRUCTURE TIDY calculated the fractional atomic coordinates to obtain the unit cells in the standardized setting. Crystal structure representations were created with DIAMOND (v. 4.6.5).^41^ Detailed information on the crystal structure investigations may be obtained from the joint CCDC/FIZ Karlsruhe (Cambridge Crystallographic Data Centre, Cambridge, UK; Fachinformationszentrum Karlsruhe, Eggenstein-Leopoldshafen, Germany) online deposition service (https://www.ccdc.cam.ac.uk/structures/) by quoting the deposition number CSD-2349766 for Sr[Li_3_AlO_4_] and CSD-2349767 for Sr[Li_3_GaO_4_].

### 2.3 Powder X-Ray Diffraction

Powders of Sr[Li_3_(Al_1–*x*_Ga_*x*_)O_4_] with the nominal gallium mole fraction *x* being equal to 0, 0.1, 0.2, 0.4, 0.6, 0.8, and 1 were investigated on an Empyrean powder diffractometer (PANalytical Xcelerator detector, Malvern Panalytical, Kassel, Germany) in the Bragg–Brentano geometry using Ge(111)-monochromatized Cu K-L_3_ radiation (λ = 1.54056 Å). The measurements were carried out in 2θ range from 10 to 80° with a step size of 0.0084° and 15 s·step^–1^. Data collection and processing were performed in HIGHSCOREPLUS (v. 4.9)^42^ including refinement of the unit-cell parameters and the phase compositions based on theoretical diffraction patterns of the respective compound in the triclinic space group *P*1̅ (no. 2).

### 2.4 Photoluminescence

The photoluminescence measurements described below were carried out on the Eu^2+^-activated compounds. The emission spectra of powder plaques of Sr[Li_3_(Al_1–*x*_Ga_*x*_)O_4_] with the nominal gallium mole fraction *x* being equal to 0, 0.1, 0.2, 0.4, 0.6, 0.8, and 1 were recorded at room temperature on a Fluoromax 4 spectrophotometer (Horiba, Kyoto, Japan) in diffuse reflection mode. A 150 W xenon arc lamp was used as the excitation source (λ_exc_ = 460 nm) and the data were processed in FLUORESSENCE (v. 3.5.1.20).^43^ Single crystals of Sr[Li_3_AlO_4_] and Sr[Li_3_GaO_4_] were excited on a microscope slide at ambient conditions using a pigtailed InGaN-based laser diode (λ_exc_ = 448 nm, Thorlabs, Ostfildern, Germany). The emission intensities were detected with a luminescence spectrometer (AvaSpec-ULS2048, Avantes, Apeldoorn, Netherlands) and the data were processed with OCEANVIEW (v. 2.0.4).^44^ The relative emission intensities of Sr[Li_3_AlO_4_] and Sr[Li_3_GaO_4_] were measured in the temperature range from 298 to 498 K with a 25 K step size (λ_exc_ = 460 nm) to study their thermal quenching behavior. Therefore, the powders were placed on a heating plate in the Fluoromax 4 spectrophotometer in form of a thin layer and the temperature was controlled using a thermocouple. Powder samples of Sr[Li_3_(Al_1–*x*_Ga_*x*_)O_4_] with the nominal gallium mole fraction *x* being equal to 0, 0.1, 0.2, and 0.6 were embedded in a silicon matrix. The emission intensities were recorded with a Quantaurus-QY spectrometer (Hamamatsu Photonics, Hamamatsu, Japan) using a 150 W xenon arc lamp (λ_exc_ = 450 nm) as the excitation source to determine the quantum efficiency Φ (integrating sphere diameter ∼8.4 cm). Data processing was performed with PLQY U6039-05 (v. 4.0.1).^45^ The luminescence decay curve of Sr[Li_3_AlO_4_] was measured at *T* = 298 K using a FLSP920 spectrometer (Edinburgh Instruments, Livingston, Scotland) equipped with a nF900 hydrogen flash lamp. For data acquisition with F980 (v. 1.4.5),^46^ the sample was excited at λ_exc_ = 440 nm and the emission was detected at λ_em_ = 570 nm.

## 3 Results and Discussion

### 3.1 Crystal Structure

**Figure 2:**
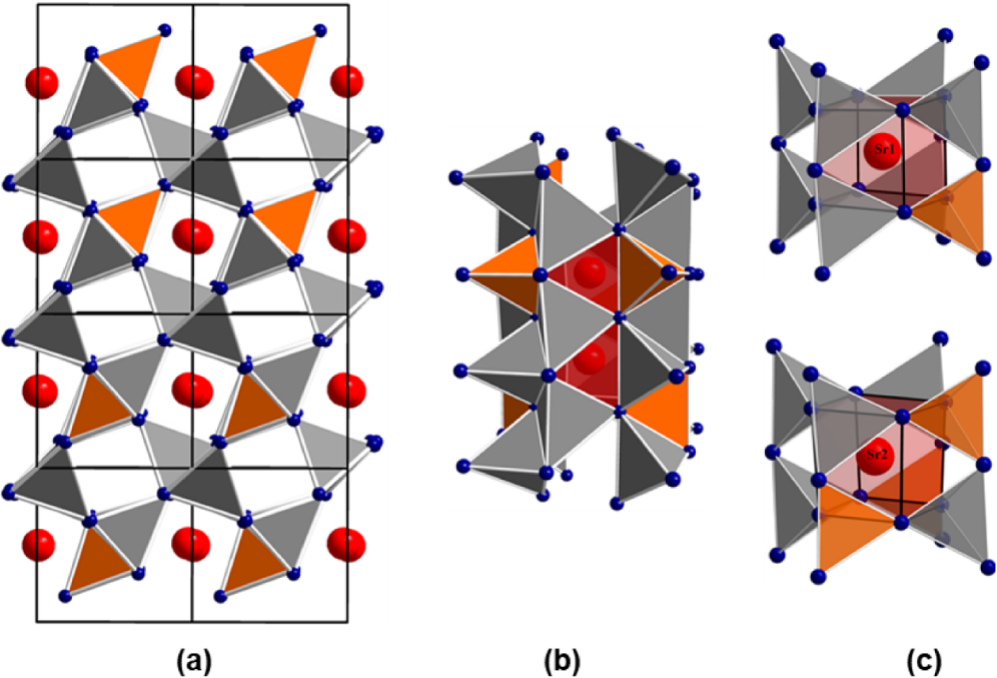
(a) [2 × 2 × 2] unit cells of Sr[Li_3_(Al/Ga)O_4_] in projection along [011] and (b) the structural section of a *vierer* ring channel formed by the arrangement of the corner- and edge-sharing tetrahedra of [(Al/Ga)O_4_] (orange) and [LiO_4_] (gray). (c) Coordination of the cube-like [SrO_8_] polyhedra (red) are shown for the two distinct strontium sites (red spheres).

Detailed information on the crystallographic data of Sr[Li_3_AlO_4_] and Sr[Li_3_GaO_4_] are summarized in Table 1. The atomic coordinates, displacement parameters, and interatomic distances are listed in Tables S2–S6. Due to the low activator concentrations of approximately 0.5 mol % used in the syntheses, the presence of europium was neglected in both structure refinements as no contribution to the X-ray scattering density was expected. Each atom is assigned to the Wyckoff position 2*i* and the structure refinements were checked for occupational and positional disorder with respect to the above-mentioned disordered average structures of the crystallites with chemical compositions close to that of Sr[Li_3_AlO_4_].^23^ The displacement parameters, interatomic distances, and site occupancy factors showed no indications for the occurrence of disordered strontium sites and no satellite reflections or irregular extinction conditions were observed in the reciprocal space to necessitate the use of modulated structure models. Sr[Li_3_AlO_4_] crystallizes in the triclinic space group *P*1̅ (no. 2) with the unit cell parameters *a* = 5.7515(2), *b* = 7.3268(2), *c* = 9.7196(3) Å, α = 83.978(1), β = 76.647(1), γ = 79.625(1)°, and a volume *V* of 391.14(2) Å^3^ measured at *T* = 296(2) K (*R*_int_ = 0.0288, *R*_σ_ = 0.0151). Our findings in the crystallographic investigation of Sr[Li_3_AlO_4_] are consistent with the data available in literature^22^ and the structural relationship to the well-known Sr[LiAl_3_N_4_]^2^ is confirmed. The graphical representation of the crystal structure is shown in Figure 2a and the structural section of a *vierer*(47) ring channel is depicted in Figure 2b. Two crystallographically distinct strontium sites are present in the crystal structure of Sr[Li_3_AlO_4_] with the second coordination spheres consisting of one aluminum and seven lithium atoms (Sr1 site) and three aluminum and five lithium atoms (Sr2 site), as shown in Figure 2c. In comparison, the second coordination spheres of the two strontium sites in Sr[LiAl_3_N_4_] are composed of five aluminum and three lithium atoms, and seven aluminum and one lithium atom, respectively. The average Sr–O distances in the [SrO_8_] polyhedra of Sr[Li_3_AlO_4_] (Ø(Sr1–O) = 2.652(2) Å, Ø(Sr2–O) = 2.683(3) Å) are shorter in length compared to the average distances in the [SrN_8_] polyhedra of Sr[LiAl_3_N_4_] (Ø(Sr1/2–N) = 2.80(2) Å), as would be expected due to the different ionic radii of the O^2–^ ion (c.n. = 4: *r*_ion_ = 1.38 Å) and the N^3–^ ion (c.n. = 4: *r*_ion_ = 1.46 Å).^48^

**Table 1:** Single-Crystal Data and Structure Refinements of Sr[Li_3_AlO_4_] and Sr[Li_3_GaO_4_] with Standard Deviations in Parentheses

empirical formula	Sr[Li_3_AlO_4_]	Sr[Li_3_GaO_4_]
rel. molar mass (g·mol^–1^)	199.42	242.16
temperature (K)	296(2)	296(2)
crystal system/space group	triclinic/*P*1̅ (no. 2)	triclinic/*P*1̅ (no. 2)
*a* (Å)	5.7515(2)	5.8204(1)
*b* (Å)	7.3268(2)	7.3846(2)
*c* (Å)	9.7196(3)	9.8034(2)
α (deg)	83.978(1)	84.185(1)
β (deg)	76.647(1)	76.768(1)
γ (deg)	79.625(1)	79.593(1)
*V* (Å^3^)	391.14(2)	402.64(2)
formula units per cell *Z*	4	4
crystal size (mm^3^)	0.040 × 0.030 × 0.010	0.050 × 0.030 × 0.020
calculated density ρ_calc_ (g·cm^–3^)	3.39	4.00
absorption coefficient μ (mm^–1^)	24.41	24.76
*F*(000) (*e*)	368	440
single-crystal diffractometer	Bruker D8 Quest	Bruker D8 Quest
radiation/wavelength (Å)	Cu K-L_2,3_/1.54178	Cu K-L_2,3_/1.54178
detector	Bruker APEX-II CCD	Bruker APEX-II CCD
absorption correction	multiscan	multiscan
2θ range (deg)	4.69–68.17	7.44–72.39
index range *hkl*	–6 ≤ *h* ≤ 6–8 ≤ *k* ≤ 8–11 ≤ *l* ≤ 11	–7 ≤ *h* ≤ 7–9 ≤ *k* ≤ 9–12 ≤ *l* ≤ 12
reflections total/independent	10567/1406	12804/1586
reflections with [*I* > 2σ(*I*)]	1323	1457
data/restraints/parameters	1406/0/164	1586/0/154
goodness-of-fit on *F*^2^	1.126	1.142
final *R*_1_/*wR*_2_ [*I* > 2σ(*I*)]	0.0211/0.0541	0.0309/0.0902
final *R*_1_/*wR*_2_ [all data]	0.0230/0.0551	0.0339/0.0929
largest diff. peak and hole (*e*Å^–3^)	0.68/–0.96	1.06/–1.27

Looking at the invidual distances, the shortest Sr–O distance is observed in the [Sr2O_8_] polyhedron of Sr[Li_3_AlO_4_] (Sr1–O: 2.564(2) Å, Sr2–O: 2.532(2) Å), while the shortest Sr–N distances are equal within the range of the standard deviations in both [SrN_8_] polyhedra of Sr[LiAl_3_N_4_] (Sr1–N: 2.69(1) Å, Sr2–N: 2.67(2) Å). A simple empirical concept to describe the degree of polyhedron distortion is given by the distortion index (*D*),^49^ which has been calculated for different types of coordination polyhedra.^50−52^ In our case, *D* is obtained by calculating the absolute deviations of the indvidual Sr–O distances from the average Sr–O distance in the [SrO_8_] polyhedron, normalized to the average Sr–O distance, and then dividing their sum by the total number of Sr–O distances. The higher degree of distortion is observed for the polyhedron of [Sr2O_8_] with a *D* value of 0.029 compared to the value of 0.019 for the [Sr1O_8_] polyhedron. The isomorphic substitution of aluminum by gallium in Sr[Li_3_AlO_4_] yields the hitherto unknown compound Sr[Li_3_GaO_4_], which is a new member of the substance class of quaternary alkaline earth lithogallates. The crystal structure of Sr[Li_3_GaO_4_] was solved and refined in the triclinic space group *P*1̅ (no. 2) with the unit cell parameters *a* = 5.8204(1), *b* = 7.3846(2), *c* = 9.8034(2) Å, α = 84.185(1), β = 76.768(1), γ = 79.593(1)°, and a volume *V* of 402.64(2) Å^3^ measured at *T* = 296(2) K (*R*_int_ = 0.0658, *R*_σ_ = 0.0343). The interatomic cation–anion distances are within the expected range as observed for representatives of strontium litho(alumo)gallates.^53,54^ The 18 crystallographically distinct sites in Sr[Li_3_GaO_4_] are occupied analogously to those described for Sr[Li_3_AlO_4_].^22^ The Ga^3+^ ions (c.n. = 4: r_ion_ = 0.47 Å) replace the Al^3+^ ions (c.n. = 4: r_ion_ = 0.39 Å),^48^ resulting in larger average Ga–O distances (Ø(Ga1–O) = 1.855(4) Å, Ø(Ga2–O) = 1.858(4) Å) compared to the average Al–O distances (Ø(Al1–O) = 1.775(3) Å, Ø(Al2–O) = 1.775(3) Å) in Sr[Li_3_AlO_4_]. The average Sr–O distances in the [SrO_8_] polyhedra of Sr[Li_3_GaO_4_] (Ø(Sr1–O) = 2.657(4) Å, Ø(Sr2–O) = 2.681(4) Å) are almost equal in length compared to the average Sr–O distances in Sr[Li_3_AlO_4_]. The shortest Sr–O distance (Sr1–O: 2.590(4) Å, Sr2–O: 2.541(4) Å) and the longest Sr–O distance (Sr1–O: 2.731(4) Å, Sr2–O: 2.889(4) Å) are found in the [Sr2O_8_] polyhedron of Sr[Li_3_GaO_4_]. The *D* value of 0.028 for the [Sr2O_8_] polyhedron is close to the value calculated for Sr[Li_3_AlO_4_], but the degree of distortion reduces for the [Sr1O_8_] polyhedron of Sr[Li_3_GaO_4_] to 0.011. In summary, the higher values of polyhedra distortion were calculated for the [Sr2O_8_] polyhedra with three aluminum atoms or gallium atoms in the second coordination sphere of the strontium atoms in Sr[Li_3_(Al/Ga)O_4_]. The shortest Sr–O distances in the [SrO_8_] polyhedra of Sr[Li_3_GaO_4_] are slightly greater in length and the longest Sr–O distance in the [Sr2O_8_] polyhedron decreases compared to the observed Sr–O distances in the crystal structure of Sr[Li_3_AlO_4_].

### 3.2 Powder X-Ray Diffraction

**Figure 3:**
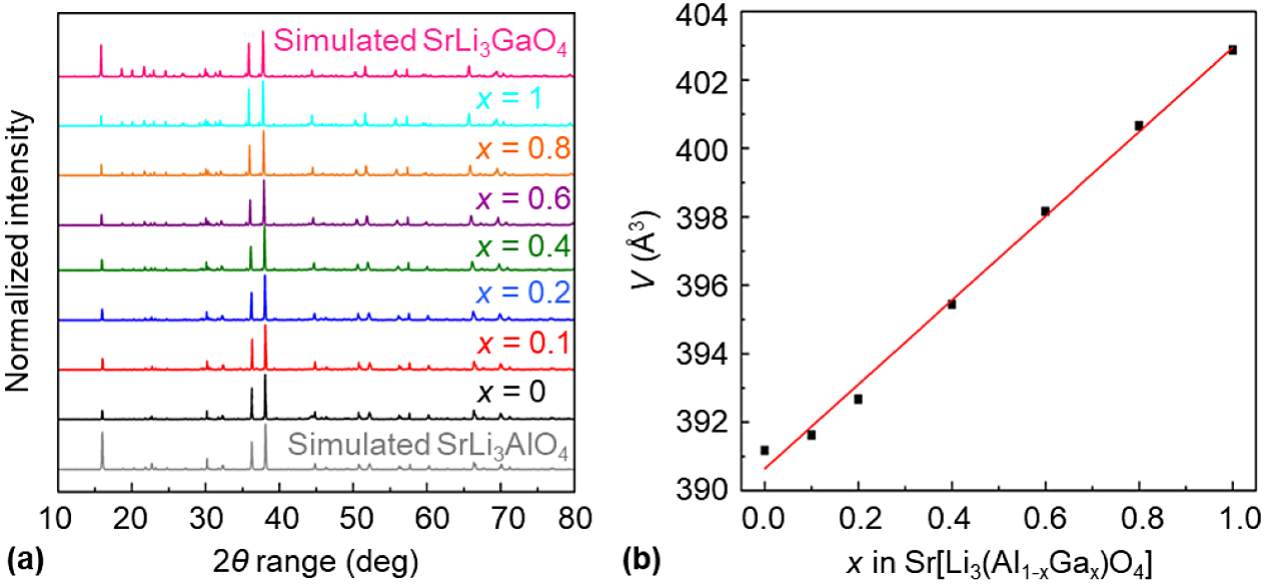
(a) Experimental X-ray diffraction patterns of seven powder samples of Sr[Li_3_(Al_1–*x*_Ga_*x*_)O_4_] with the nominal gallium mole fraction *x* being equal to 0 (black), 0.1 (red), 0.2 (blue), 0.4 (green), 0.6 (purple), 0.8 (brown), and 1 (turquoise). The simulated X-ray diffraction patterns of Sr[Li_3_AlO_4_] (gray) and Sr[Li_3_GaO_4_] (pink) were calculated from the single-crystal data. (b) Unit-cell volumes (black squares) as a function of the nominal gallium mole fraction *x* in Sr[Li_3_(Al_1–*x*_Ga_*x*_)O_4_]. *V* is derived from the Rietveld refined unit-cell parameters obtained from powder X-ray diffraction data (Cu K-L_3_ radiation, λ = 1.54056 Å) and the straight line (red) represents the linear regression of the variables shown.

The normalized intensities of the experimental X-ray diffraction patterns of seven powder samples of Sr[Li_3_(Al_1–*x*_Ga_*x*_)O_4_] are shown in Figure 3a together with the theoretical X-ray diffraction patterns of Sr[Li_3_AlO_4_] and Sr[Li_3_GaO_4_] (based on the above-mentioned single-crystal X-ray diffraction data). The reflection positions and intensity ratios in the experimental X-ray diffraction patterns match those of the theoretical X-ray diffraction patterns, showing that the respective compounds were obtained as products in the solid-state syntheses in nickel crucibles. As expected, changes in the intensity distributions can be observed in the experimental X-ray diffraction patterns, when the gallium mole fraction increases. The refined unit-cell parameters as a function of the nominal gallium mole fraction *x* in Sr[Li_3_(Al_1–*x*_Ga_*x*_)O_4_] are listed in Table S7. The graphical representations of the changes in the individual parameters are shown in Figure S1. The unit-cell parameters obtained from single-crystal and powder X-ray diffraction data are in agreement, leading us to conclude that the structure model of Sr[Li_3_(Al/Ga)O_4_] derived from single-crystal data is representative for the powder samples. The Rietveld refinements of six selected powder samples are depicted in Figure S2. The experimental X-ray diffraction patterns are explained by the presence of Sr[Li_3_(Al_1–*x*_Ga_*x*_)O_4_] in the corresponding composition, which shows that the target compounds in the solid-solution series were obtained as main products. Secondary phases with minor phase fractions are also present in the powder samples. SrLiBO_3_ was formed by the reaction of SrO with the mineralizing agent Li_2_B_4_O_7_, and Sr_2_[Li(Al_1–*x*_Ga_*x*_)O_4_] was obtained from the reaction of the starting materials, exhibiting the same elements as the title compounds. After clarifying that the powder samples are representative for the compounds elucidated by single-crystal X-ray diffraction, we further investigated the solid-solution series of Sr[Li_3_(Al_1–*x*_Ga_*x*_)O_4_]. Checking the presence of Vegard behavior is one way to demonstrate that a solid-solutions series with unlimited miscibility exists, which allows us to attribute isomorphism to the title compounds. After deviations in the linear relationship between the unit-cell parameters and the concentrations of the solid-solution constituents were observed in various systems, the so-called Vegard’s law was recapitulated and proposed as an approximation valid for ideal solid solutions when the difference in the unit-cell parameters of the pure compounds is less than 5%.^55^ In Figure 3b, the unit-cell volumes are plotted against the nominal gallium mole fraction *x* in Sr[Li_3_(Al_1–*x*_Ga_*x*_)O_4_]. As expected from the obtained unit-cell parameters of Sr[Li_3_AlO_4_] and Sr[Li_3_GaO_4_], which do not differ by more than 3%, we observe an ideal Vegard behavior in the solid-solution series of Sr[Li_3_(Al_1–*x*_Ga_*x*_)O_4_] and therefore the crystal structures can be described as isomorphous.

### 3.3 Photoluminescence

**Figure 4:**
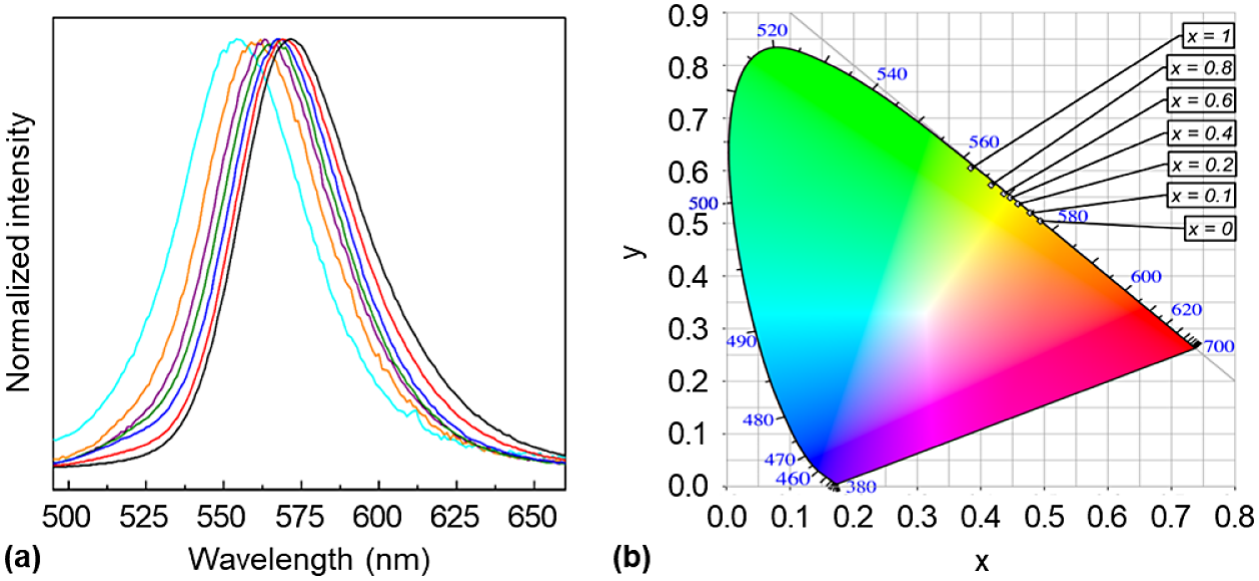
(a) Normalized emission spectra of Eu^2+^-activated Sr[Li_3_(Al_1–*x*_Ga_*x*_)O_4_] with the nominal gallium mole fraction *x* equals 0 (black), 0.1 (red), 0.2 (blue), 0.4 (green), 0.6 (purple), 0.8 (brown) and 1 (turquoise), excited with a xenon arc lamp (λ_exc_ = 460 nm). (b) Color points of Eu^2+^-activated powders of Sr[Li_3_(Al_1–*x*_Ga_*x*_)O_4_] with the nominal gallium mole fraction *x* equals 0, 0.1, 0.2, 0.4, 0.6, 0.8, and 1 in the CIE 1931 color space chromaticity diagram.

The normalized emission spectra of the Eu^2+^-activated powder samples of Sr[Li_3_(Al_1–*x*_Ga_*x*_)O_4_] are shown in Figure 4a and details on the spectral data are summarized in Table 2. The tabulated color point coordinates are depicted in the CIE 1931 color space^56^ in Figure 4b. The phosphors show green-to-yellow photoluminescence and can be effectively excited with UV to blue light, as desired for the development of pc-LEDs.^57^ Yellow-emitting Sr[Li_3_AlO_4_]:Eu^2+^ exhibits a peak wavelength at λ_em_ = 572 nm (fwhm equals 47 nm, 1446 cm^–1^, 0.18 eV), which is consistent with the emission data published in the literature.^20−22^ The spectral position of the emission band can be tuned in Eu^2+^-activated Sr[Li_3_(Al_1–*x*_Ga_*x*_)O_4_] by shifting the emission maximum to higher energies with increasing gallium mole fraction *x*, reaching a peak wavelength at λ_em_ = 554 nm (fwhm equals 49 nm, 1589 cm^–1^, 0.20 eV) for green-emitting Sr[Li_3_GaO_4_]:Eu^2+^. The tunability of the spectral position of the emission band supports the claim of isomorphism by indicating the successful incorporation of gallium into the crystal structure in the solid-solution series of Eu^2+^-activated Sr[Li_3_(Al_1–*x*_Ga_*x*_)O_4_]. As reviewed by G. Li and colleagues for various inorganic host structures, the influence of cationic substitution on the emission shift of the 4f^6^5d^1^ → 4f^7^ transitions of rare-earth ions includes different parameters that effect the nature of the activator’s environment.^24^ As a result of the cationic substitution of aluminum by gallium, the peak wavelength is shifted to higher energies, which is mainly caused by the weakening of the crystal field strength due to the less pronounced Eu-ligand interaction. Based on the single-crystal X-ray diffraction data of the boundary components of Sr[Li_3_(Al_1–*x*_Ga_*x*_)O_4_] with *x* = 0 and 1, the slightly larger shortest Sr/Eu–O distances in combination with a reduced polyhedron distortion, which means less structure relaxation around the activator in its excited state, the reduced crystal field splitting of the 5d-orbitals causes the shift of the emission maximum to higher energies. The observed emission bands of Eu^2+^-activated Sr[Li_3_(Al_1–*x*_Ga_*x*_)O_4_] are located at higher energies compared to the isotypic compound Sr[LiAl_3_N_4_]:Eu^2+^ with a peak wavelength at approximately λ_em_ = 654 nm (fwhm equals 50 nm, 1180 cm^–1^, 0.146 eV).^2^

**Table 2:** Spectral Data of the Eu^2+^-Activated Powder Samples of Sr[Li_3_(Al_1–*x*_Ga_*x*_)O_4_] Showing the Nominal Gallium Mole Fraction *x*, Peak Wavelength λ_em_ (nm), Dominant Wavelength λ_dom_ (nm), and the CIE-xy Values in the CIE 1931 Color Space

*x*	λ_em_ (nm)	λ_dom_ (nm)	CIE-x	CIE-y
0	572	577	0.493(1)	0.504(1)
0.1	570	575	0.477(1)	0.519(1)
0.2	568	572	0.458(1)	0.536(1)
0.4	566	570	0.446(1)	0.548(1)
0.6	564	569	0.436(1)	0.556(1)
0.8	560	566	0.416(1)	0.572(1)
1.0	554	562	0.384(1)	0.604(1)

The new phosphors show emissions at even higher energies as described for Sr[Li_2_Al_2_O_2_N_2_]:Eu^2+^ (λ_em_ = 614 nm, fwhm equals 48 nm, 1286 cm^–1^, 0.159 eV),^5^ which is also an ordered variant of the U[Cr_4_C_4_]-type structure with comparable Sr–N/O distances as in Sr[Li_3_(Al/Ga)O_4_] and Sr[LiAl_3_N_4_]. The shift of spectral position of the emission band to higher energies can be explained by the reduced nephelauxetic effect regarding oxygen ligands compared to nitrogen ligands in the activators’ coordination sphere, since the 4f^6^5d^1^ ↔ 4f^7^ transitions of Eu^2+^ are highly sensitive to the local chemical environment. In the crystal structure of Sr[LiAl_3_N_4_]:Eu^2+^, only Eu–N bonds are present and the covalency reduces for the Eu–N/O bonds in Sr[Li_2_Al_2_O_2_N_2_]:Eu^2+^ to exclusively Eu–O bonds in Sr[Li_3_(Al_1–*x*_Ga_*x*_)O_4_]:Eu^2+^. The value of the full width at half-maximum (fwhm) increases on the energy proportional scales at room temperature. Sr[LiAl_3_N_4_]:Eu^2+^ (1180 cm^–1^, 0.146 eV) exhibits the smallest value, which increases from Sr[Li_2_Al_2_O_2_N_2_]:Eu^2+^ (1286 cm^–1^, 0.159 eV) to Sr[Li_3_AlO_4_]:Eu^2+^ (1446 cm^–1^, 0.18 eV) with the highest one found for Sr[Li_3_GaO_4_]:Eu^2+^ (1589 cm^–1^, 0.20 eV).

**Figure 5:**
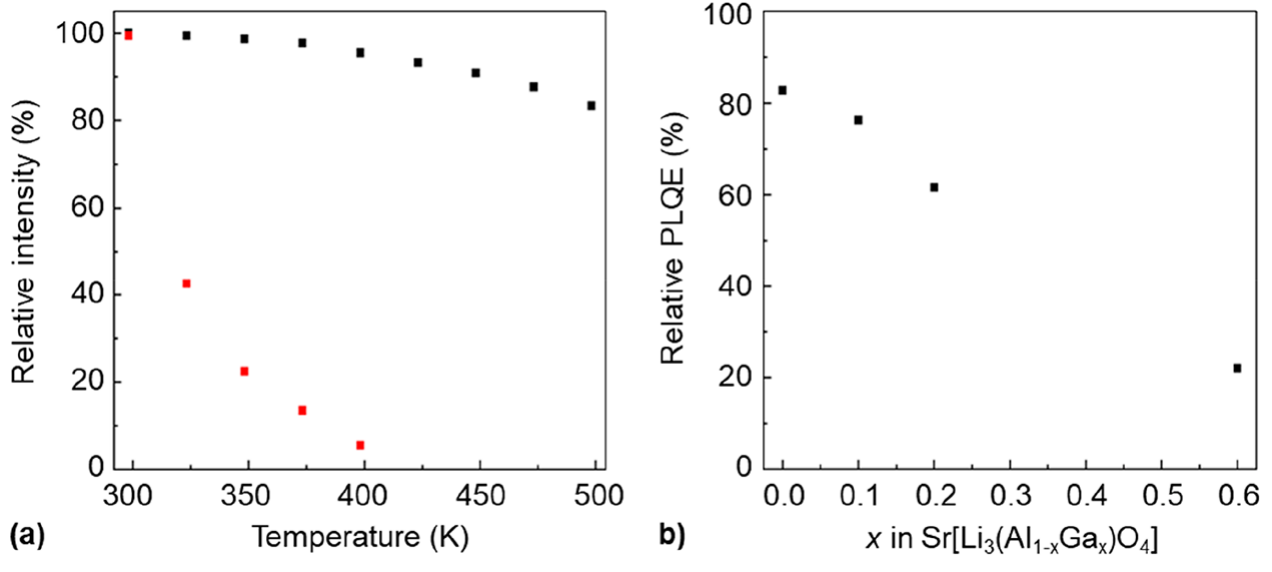
(a) Thermal quenching resistance of Sr[Li_3_AlO_4_]:Eu^2+^ (black squares) and Sr[Li_3_GaO_4_]:Eu^2+^ (red squares) powder samples showing the integrated photoluminescence emission intensities (λ_exc_ = 460 nm) relative to the intensity at *T* = 298 K. The temperature was varied in the range from 298 to 498 K with steps of 25 K. (b) Relative photoluminescence quantum efficiency (PLQE) of Eu^2+^-activated powder samples of Sr[Li_3_(Al_1–*x*_Ga_*x*_)O_4_] (black squares) with *x* equals 0, 0.1, 0.2, and 0.6 excited with blue light (λ_exc_ = 450 nm).

We assume that Eu^2+^ is distributed on two crystallographically distinct sites in Sr[Li_3_(Al/Ga)O_4_]. A comparable situation exisits for Sr[LiAl_3_N_4_]:Eu^2+^, where time-resolved and low-temperature luminescence spectroscopy at *T* = 10 K were used to decompose the virbronic structure of the emission transitions, revealing that Eu^2+^ occupies two crystallographically distinct strontium sites.^8,9^ The luminescence of the single crystals used for the structure determinations of the title compounds were investigated and the normalized emission spectra of the Eu^2+^-activated compounds are shown in Figure S3. Peak wavelengths were observed at λ_em_ = 568 nm with a fwhm equals 46 nm for Sr[Li_3_AlO_4_]:Eu^2+^ and at λ_em_ = 552 nm with a fwhm equals 48 nm for Sr[Li_3_GaO_4_]:Eu^2+^. The single-crystal emission data agree very well with the emission data of the bulk samples. We conclude that the emissions of the Eu^2+^-activated powder samples are represented by the single-crystal emissions of Sr[Li_3_(Al/Ga)O_4_]:Eu^2+^. These spectra measured on single crystals are the best representatives for the emission of the Eu^2+^-activated title compounds, as their spectra are not subject to reabsorption effects. These reabsorption effects are also most likely the cause for the minor deviations from the powder sample data. In order to assign the experimental emission spectra to the 4f^6^5d^1^ → 4f^7^ transitions of Eu^2+^, the luminescence decay curve of Sr[Li_3_AlO_4_]:*x*Eu^2+^ (*x* = 0.5 mol % nominal concentration) was measured at room temperature, which is shown in Figure S6. The luminescence decay is single exponential with a radiative decay time of τ = (0.916 ± 0.005) μs. As observed for other inorganic phosphors with the U[Cr_4_C_4_]-type structure such as Sr[Li_2_Al_2_O_2_N_2_]:Eu^2+^ (τ = 0.86 ± 0.01 μs at *T* = 298 K),^11^ the value of τ is within the range expected for 4f^6^5d^1^ ↔ 4f^7^ transitions of Eu^2+^. Although no 4f → 4f emission transitions attributable to the spin–orbit levels of the Eu^3+^ ion and the associated crystal-field sublevels were observed in the emission spectra, the presence of Eu^3+^ ions that were not reduced to Eu^2+^ under the applied synthesis conditions cannot be ruled out. Regarding the thermal quenching behavior, the requirement for an (inorganic) phosphor is that the photoluminescence emission intensity decreases only slightly up to an LED operating temperature of approximately 423 K relative to the emission intensity at room temperature.^58^ In Figure 5a, the temperature-dependent emission intensities of the powder samples of Sr[Li_3_AlO_4_]:Eu^2+^ and Sr[Li_3_GaO_4_]:Eu^2+^ are shown in the temperature range from 298 to 498 K. The integrated photoluminescence intensity of the emission spectrum of Sr[Li_3_AlO_4_]:Eu^2+^ decreases to 83% within the measured temperature range, revealing excellent thermal quenching resistance with an emission intensity of >93% at *T* = 423 K relative to the value at room temperature. The thermal quenching resistance of Sr[Li_3_AlO_4_]:Eu^2+^ is slightly weaker than that for Sr[Li_2_Al_2_O_2_N_2_]:Eu^2+^ with a relative photoluminescence emission intensity of >96% at *T* = 420 K.^5^ Even superior thermal quenching resistance was reported for isotypically crystallizing Sr[LiAl_3_N_4_]:Eu^2+^, retaining >95% of the relative photoluminescence emission intensity at *T* = 500 K.^2^ Sr[Li_3_GaO_4_]:Eu^2+^ exhibits a poor thermal quenching resistance with an integrated photoluminescence emission intensity of 5% at *T* = 398 K relative to the room temperature value. The temperature-dependent photoluminescence emission spectra are presented in Figures S4 and S5. The emission band of Sr[Li_3_AlO_4_]:Eu^2+^ is shifted to higher energies and its shape becomes more asymmetric on the high-energy side of the spectrum with increasing temperature. This temperature behavior of the emission spectrum is already known from structurally related Sr[Li_2_Al_2_O_2_N_2_]:Eu^2+^.^5^

The UV-irradiated powder samples of Eu^2+^-activated Sr[Li_3_(Al_1–*x*_Ga_*x*_)O_4_] in Figure 1 show that the replacement or partial substitution of aluminum by gallium lead to a decrease in the photoluminescence emission intensity with increasing gallium mole fraction, which is already perceptible to the human eye. The qualitative trend is experimentally confirmed by the measured photoluminescence quantum efficiency Φ of four selected powder samples under blue-light excitation, as shown in Figure 5b. Relative quantum efficiencies of Sr[Li_3_(Al_1–*x*_Ga_*x*_)O_4_]:Eu^2+^ were obtained for *x* = 0 (Φ = 83%), *x* = 0.1 (Φ = 76%) and *x* = 0.2 (Φ = 62%). To confirm the decrease of the quantum efficiency for a gallium-rich solid solution of Sr[Li_3_(Al_1–*x*_Ga_*x*_)O_4_]:Eu^2+^ (*x* > 0.5), a measurement was performed that yielded a quantum efficiency of Φ = 22% for the nominal gallium mole fraction *x* = 0.6. The missing values of Φ for the members of the solid-solution series are due to the presence of probably absorbing or scattering impurities and secondary phases caused by the material of the crucibles and/or the starting materials used in the syntheses.

## 4 Conclusion

The new quaternary compound Sr[Li_3_GaO_4_] was synthesized at moderate temperature, demonstrating the successful replacement of aluminum by gallium in Sr[Li_3_AlO_4_]. Single-crystal and powder X-ray diffraction analysis revealed the presence of a solid-solution series in Sr[Li_3_(Al_1–*x*_Ga_*x*_)O_4_] and the isomorphic crystallization of the oxolithoaluminate and the oxolithogallate was confirmed. In a first approximation and under the assumption that Eu^2+^ occupies the two crystallographically distinct strontium sites in Sr[Li_3_(Al/Ga)O_4_], the position of the emission band is presumable related to the bonding situation within the cation polyhedra. Although the average Sr–O distances of the [SrO_8_] polyhedra are almost equal in Sr[Li_3_AlO_4_] and Sr[Li_3_GaO_4_], the shortest Sr–O distances are slightly longer in the oxolithogallate compared to the oxolithoaluminate. In addition, the polyhedra distortion is lower in the oxolithogallate as in the oxolithoaluminate. Both, the reduced crystal-field splitting and the lower polyhedra distortion in the oxolithogallate would cause a slight shift of the emission band to higher energies (lower wavelenghts). Sr[Li_3_AlO_4_]:Eu^2+^ exhibits potential as an inorganic phosphor for solid-state lighting applications due to its high quantum efficiency and excellent thermal quenching behavior, while both properties deteriorate significantly for the Eu^2+^-activated gallium-containing derivates and the oxolithogallate, respectively. A possible explanation for the reduction in quantum yield with increasing gallium content could be the different thermal behavior. The thermal quenching data for Sr[Li_3_AlO_4_]:Eu^2+^ suggest only minimal thermal quenching at room temperature. The significantly worse thermal behavior for Sr[Li_3_GaO_4_]:Eu^2+^, however, could hint at significant thermal losses already at room temperature. We thus hypothesize, that the reduction in quantum yield with increasing gallium mole fraction *x* in Sr[Li_3_(Al_1–*x*_Ga_*x*_)O_4_]:Eu^2+^ is mainly caused by increased thermal quenching at room temperature. Due to the structural relationship of the presented oxolitho(alumo)gallates with already known and intensively studied U[Cr_4_C_4_]-type phosphors, future investigations involving time-resolved and low-temperature luminescence spectroscopy in combination with first-principles calculations could be of interest to develop a deeper understanding of the Eu(II) luminescence as a function of the composition of the host structure.

## Supporting Information Available

Stoichiometric ratios used in the solid-state syntheses (Table S1); atomic coordinates, displacement parameters, and interatomic distances of Sr[Li_3_AlO_4_] and Sr[Li_3_GaO_4_] derived from single-crystal X-ray diffraction (Tables S2–S6); tabular (Table S7) and graphical (Figure S1) representations of the refined unit-cell parameters including the Rietveld refinements (Figure S2) of Sr[Li_3_(Al_1–*x*_Ga_*x*_)O_4_] derived from powder X-ray diffraction; normalized emission spectra of Eu^2+^-activated Sr[Li_3_(Al/Ga)O_4_] single crystals (Figure S3); temperature-dependent photoluminescence spectra of Eu^2+^-activated powder samples of Sr[Li_3_(Al/Ga)O_4_] (Figures S4 and S5); room temperature luminescence decay curve of Sr[Li_3_AlO_4_]:Eu^2+^ (Figure S6) (PDF)
