# Supplementary material for: Isomorphism of Sr[Li3AlO4] and Sr[Li3GaO4] – Syntheses, Crystal Structure, and Europium(II) Luminescence
Source: Chem Mater. 2024 Jul 18;36(15):7429–37. doi: 10.1021/acs.chemmater.4c01382 (PMC11325535; doi:10.1021/acs.chemmater.4c01382)
Supplement: Supplementary file 1 — cm4c01382_si_001.pdf [file cm4c01382_si_001.pdf]

Supporting Information

Isomorphism of $Sr[Li_3AlO_4]$ and $Sr[Li_3GaO_4]$ – syntheses, crystal structure, and europium(II) luminescence

Johannes G. Volpini[†], *Mark Vorsthove*[‡], *Christiane Stoll*[‡], *Daniel Bichler*[‡], *Markus Seibald*[‡], *and Hubert Huppertz*[†,*]

[†]Department of General, Inorganic and Theoretical Chemistry, University of Innsbruck, Innrain 80-82, AT-6020 Innsbruck, Austria,

[‡]ams-OSRAM International GmbH, Mittelstetter Weg 2, D-86830 Schwabmünchen, Germany

TABLE OF CONTENT

A: SYNTHESIS

Stoichiometric ratios of the starting materials used in the solid-state syntheses to obtain powder samples of $Sr[Li_3(Al_{1-x}Ga_x)O_4]$.

B: CRYSTAL STRUCTURE

Atomic coordinates, equivalent isotropic/anisotropic displacement parameters, and interatomic distances of $Sr[Li_3AlO_4]$ and $Sr[Li_3GaO_4]$.

C: POWDER X-RAY DIFFRACTION

Tabular and graphical representations of the refined unit-cell parameters as a function of the nominal gallium mole fraction *x* in $Sr[Li_3(Al_{1-x}Ga_x)O_4]$. Rietveld refinements of six bulk samples.

D: PHOTOLUMINESCENCE

Normalized emission spectra of $Sr[Li_3AlO_4]:Eu^{2+}$ and $Sr[Li_3GaO_4]:Eu^{2+}$ single crystals. Temperature-dependent photoluminescence spectra of the Eu^{2+}-activated powder samples of $Sr[Li_3(Al/Ga)O_4]$. Luminescence decay curve of $Sr[Li_3AlO_4]:Eu^{2+}$.

A: SYNTHESIS

Table S1: Stoichiometric ratios of the starting materials used in the solid-state syntheses of $Sr[Li_3(Al_{1-x}Ga_x)O_4]$ with the nominal gallium mole fraction *x* being equal to 0, 0.1, 0.2, 0.4, 0.6, 0.8, and 1.

x	m(SrO) / g	m($Sr_3Al_2N_4$) / g	m($SrAl_2O_4$) / g	m(Li_2O) / g	m(Ga_2O_3) / g	m(GaN) / g
0	4.13	1.86	9.21	4.46	-	-
0.1	4.54	1.82	8.01	4.37	0.91	-
0.2	4.95	1.78	6.87	4.28	1.79	-
0.4	5.70	1.71	4.71	4.11	3.44	-
0.6	6.40	1.65	2.72	3.96	4.96	-
0.8	7.05	1.59	0.87	3.81	6.38	-
1	8.47	-	-	3.66	-	0.68
x	n(SrO) / mol	n($Sr_3Al_2N_4$) / mol	n($SrAl_2O_4$) / mol	n(Li_2O) / mol	n(Ga_2O_3) / mol	n(GaN) / mol
0	0.0399	0.0050	0.0448	0.1493	-	-
0.1	0.0438	0.0049	0.0390	0.1463	0.0049	-
0.2	0.0478	0.0048	0.0334	0.1432	0.0096	-
0.4	0.0550	0.0046	0.0229	0.1376	0.0184	-
0.6	0.0618	0.0044	0.0132	0.1325	0.0265	-
0.8	0.0680	0.0043	0.0042	0.1275	0.0340	-
1	0.0817	-	-	0.1225	-	0.0081

B: CRYSTAL STRUCTURE

Table S2: Atomic coordinates and equivalent isotropic displacement parameters U_{eq} (Å^2) for Sr[Li_3AlO_4] with standard deviations in parentheses. Each crystallographic site is assigned to the Wyckoff position 2*i*.

Site	*x*	*y*	*z*	U_{eq}
Al1	0.4588(2)	0.7881(2)	0.1344(1)	0.0047(2)
Al2	0.8391(2)	0.3033(2)	0.3459(1)	0.0044(2)
Li1	0.197(2)	0.1724(9)	0.1475(7)	0.014(2)
Li2	0.196(2)	0.4421(9)	0.4012(6)	0.011(2)
Li3	0.448(2)	0.0497(9)	0.3727(7)	0.011(2)
Li4	0.476(2)	0.3215(9)	0.6134(7)	0.010(2)
Li5	0.534(2)	0.4290(9)	0.1281(7)	0.012(2)
Li6	0.815(2)	0.0622(9)	0.0995(7)	0.016(2)
Sr1	0.00896(6)	0.62022(4)	0.11648(3)	0.0116(2)
Sr2	0.03198(6)	0.13469(4)	0.62468(3)	0.0090(2)
O1	0.1385(4)	0.1893(3)	0.3575(3)	0.0068(5)
O2	0.1731(4)	0.4695(3)	0.6045(3)	0.0071(5)
O3	0.1904(4)	0.9257(3)	0.0997(3)	0.0071(5)
O4	0.3322(4)	0.6157(3)	0.2602(2)	0.0067(5)
O5	0.3584(4)	0.1167(3)	0.7775(3)	0.0073(5)
O6	0.3568(4)	0.3399(3)	0.0067(3)	0.0075(5)
O7	0.6553(4)	0.1604(3)	0.4632(3)	0.0084(5)
O8	0.8401(4)	0.3044(3)	0.1633(2)	0.0065(5)

Table S3: Anisotropic displacement parameters U_{ij} (Å^2) for Sr[Li_3AlO_4] with standard deviations in parentheses.

Site	U_{11}	U_{22}	U_{33}	U_{23}	U_{13}	U_{12}
Al1	0.0047(5)	0.0050(5)	0.0041(5)	-0.0006(4)	-0.0004(4)	-0.0007(4)
Al2	0.0045(5)	0.0046(5)	0.0040(5)	-0.0004(4)	-0.0009(4)	-0.0006(4)
Li1	0.015(3)	0.011(3)	0.014(3)	0	0.002(2)	-0.005(2)
Li2	0.014(3)	0.010(3)	0.009(3)	0.002(2)	0	-0.006(2)
Li3	0.006(3)	0.016(3)	0.012(3)	-0.004(2)	0	-0.002(2)
Li4	0.007(3)	0.013(3)	0.012(3)	-0.002(2)	-0.004(2)	0
Li5	0.010(3)	0.011(3)	0.014(3)	-0.003(2)	-0.004(2)	0.001(2)
Li6	0.024(4)	0.013(3)	0.014(3)	0.002(3)	-0.006(3)	-0.008(3)
Sr1	0.0104(2)	0.0126(2)	0.0124(2)	0	-0.0032(2)	-0.0029(2)
Sr2	0.0094(2)	0.0085(2)	0.0075(2)	0	0.0001(2)	0.0004(2)
O1	0.005(2)	0.008(2)	0.007(2)	-0.0004(9)	-0.0018(9)	0.0019(9)
O2	0.007(2)	0.007(2)	0.007(2)	-0.0020(9)	-0.0024(9)	0.0001(9)
O3	0.007(2)	0.007(2)	0.008(2)	-0.0014(9)	-0.0024(9)	-0.0005(9)
O4	0.007(2)	0.008(2)	0.006(2)	0.0003(9)	-0.0009(9)	-0.0029(9)
O5	0.008(2)	0.008(2)	0.007(2)	-0.0008(9)	-0.0017(9)	-0.0043(9)
O6	0.007(2)	0.008(2)	0.007(2)	-0.0017(9)	0.0003(9)	-0.0025(9)
O7	0.009(2)	0.009(2)	0.007(2)	-0.0008(9)	0.0001(9)	-0.003(1)
O8	0.007(2)	0.007(2)	0.005(2)	-0.0010(9)	-0.0011(9)	-0.0011(9)

Table S4: Atomic coordinates and equivalent isotropic displacement parameters U_{eq} ($Å^2$) for $Sr[Li_3GaO_4]$ with standard deviations in parentheses. Each crystallographic site is assigned to the Wyckoff position 2*i*.

Site	*x*	*y*	*z*	U_{eq}
Ga1	0.4632(2)	0.79380(9)	0.13176(7)	0.0042(2)
Ga2	0.8329(2)	0.30694(9)	0.34619(7)	0.0046(2)
Li1	0.194(2)	0.178(2)	0.149(1)	0.010(2)
Li2	0.199(2)	0.442(2)	0.401(2)	0.014(2)
Li3	0.452(2)	0.048(2)	0.373(2)	0.010(2)
Li4	0.471(2)	0.313(2)	0.616(1)	0.008(2)
Li5	0.530(2)	0.433(2)	0.131(2)	0.008(2)
Li6	0.820(2)	0.060(2)	0.100(2)	0.014(2)
Sr1	0.01001(8)	0.62237(6)	0.11785(5)	0.0069(2)
Sr2	0.02633(9)	0.13303(6)	0.62288(5)	0.0070(2)
O1	0.1451(7)	0.1874(5)	0.3579(4)	0.0079(7)
O2	0.1713(7)	0.4580(5)	0.6045(4)	0.0079(8)
O3	0.1831(7)	0.9318(5)	0.0990(4)	0.0085(8)
O4	0.3311(6)	0.6149(5)	0.2636(4)	0.0062(7)
O5	0.3461(7)	0.1144(5)	0.7742(4)	0.0071(7)
O6	0.3470(7)	0.3452(5)	0.0111(4)	0.0069(7)
O7	0.6529(7)	0.1547(5)	0.4692(4)	0.0084(8)
O8	0.8382(7)	0.3071(5)	0.1563(4)	0.0074(7)

Table S5: Anisotropic displacement parameters U_{ij} (Å^2) for Sr[Li_3GaO_4] with standard deviations in parentheses.

Site	U_{11}	U_{22}	U_{33}	U_{23}	U_{13}	U_{12}
Ga1	0.0036(3)	0.0049(3)	0.0040(3)	0.0001(2)	-0.0005(2)	-0.0009(2)
Ga2	0.0052(3)	0.0041(3)	0.0043(4)	0.0008(2)	-0.0011(3)	-0.0005(2)
Li1	0.015(4)	0.010(5)	0.007(5)	-0.003(4)	0	-0.005(4)
Li3	0.003(4)	0.007(4)	0.023(6)	-0.007(4)	-0.004(4)	-0.001(3)
Li5	0.005(4)	0.009(4)	0.013(5)	-0.006(4)	-0.007(4)	0.003(3)
Li6	0.023(5)	0.008(4)	0.011(5)	0.001(4)	-0.008(4)	0.003(4)
Sr1	0.0070(3)	0.0073(3)	0.0069(3)	-0.0006(2)	-0.0021(2)	-0.0020(2)
Sr2	0.0084(3)	0.0067(3)	0.0054(3)	-0.0003(2)	-0.0011(2)	-0.0007(2)
O1	0.008(2)	0.008(2)	0.007(2)	0	-0.001(2)	0.001(2)
O2	0.007(2)	0.009(2)	0.008(2)	-0.001(2)	-0.003(2)	-0.001(2)
O3	0.007(2)	0.009(2)	0.010(2)	-0.001(2)	-0.004(2)	0.001(2)
O4	0.005(2)	0.010(2)	0.004(2)	0.001(2)	0.001(2)	-0.004(2)
O5	0.007(2)	0.007(2)	0.009(2)	0.002(2)	-0.002(2)	-0.005(2)
O6	0.007(2)	0.009(2)	0.006(2)	0.001(2)	-0.001(2)	-0.004(2)
O7	0.012(2)	0.008(2)	0.005(2)	0	0.001(2)	-0.007(2)
O8	0.008(2)	0.006(2)	0.009(2)	0.002(2)	-0.004(2)	-0.001(2)

Table S6: Interatomic distances in Sr[Li_3AlO_4] and Sr[Li_3GaO_4] obtained from single-crystal X-ray diffraction with standard deviations in parentheses.

Compound	Sr[Li_3AlO_4]		Sr[Li_3GaO_4]	
	Bond	Distance (Å)	Bond	Distance (Å)
	Al1–O3	1.767(3)	Ga1–O3	1.837(4)
	Al1–O4	1.793(3)	Ga1–O4	1.891(4)
	Al1–O5	1.770(2)	Ga1–O5	1.846(4)
	Al1–O6	1.768(3)	Ga1–O6	1.846(4)
	**Ø(Al1–O)**	**1.775(3)**	**Ø(Ga1–O)**	**1.855(4)**
	Al2–O1	1.797(3)	Ga2–O1	1.894(4)
	Al2–O2	1.766(3)	Ga2–O2	1.843(4)
	Al2–O7	1.762(3)	Ga2–O7	1.840(4)
	Al2–O8	1.773(3)	Ga2–O8	1.855(4)
	**Ø(Al2–O)**	**1.775(3)**	**Ø(Ga2–O)**	**1.858(4)**
	Li1–O1	2.004(7)	Li1–O1	2.01(1)
	Li1–O3	1.922(7)	Li1–O3	1.94(1)
	Li1–O6	1.922(7)	Li1–O6	1.92(2)
	Li1–O8	2.085(7)	Li1–O8	2.11(2)
	**Ø(Li1–O)**	**1.983(7)**	**Ø(Li1–O)**	**2.00(2)**
	Li2–O1	2.042(7)	Li2–O1	2.06(2)
	Li2–O2	2.116(7)	Li2–O2	1.98(2)
	Li2–O2	1.979(7)	Li2–O2	2.16(2)
	Li2–O4	1.899(6)	Li2–O4	1.89(2)
	**Ø(Li2–O)**	**2.009(7)**	**Ø(Li2–O)**	**2.02(2)**
	Li3–O1	1.913(7)	Li3–O1	1.92(2)
	Li3–O5	1.999(7)	Li3–O5	1.99(2)

Compound	$Sr[Li_3AlO_4]$		$Sr[Li_3GaO_4]$	
	Bond	Distance (Å)	Bond	Distance (Å)
	Li3–O7	2.153(7)	Li3–O7	2.10(2)
	Li3–O7	1.961(6)	Li3–O7	1.97(2)
	**Ø(Li3–O)**	**2.007(7)**	**Ø(Li3–O)**	**2.00(2)**
	Li4–O2	1.894(6)	Li4–O2	1.90(2)
	Li4–O4	1.965(6)	Li4–O4	1.99(1)
	Li4–O5	2.149(7)	Li4–O5	2.12(1)
	Li4–O7	1.953(7)	Li4–O7	1.94(1)
	**Ø(Li4–O)**	**1.990(7)**	**Ø(Li4–O)**	**1.99(2)**
	Li5–O4	1.994(7)	Li5–O4	1.99(1)
	Li5–O6	1.953(7)	Li5–O6	1.98(1)
	Li5–O6	2.125(7)	Li5–O6	2.16(2)
	Li5–O8	1.919(7)	Li5–O8	1.92(1)
	**Ø(Li5–O)**	**1.998(7)**	**Ø(Li5–O)**	**2.01(2)**
	Li6–O3	2.211(7)	Li6–O3	2.15(2)
	Li6–O3	1.936(7)	Li6–O3	1.95(2)
	Li6–O5	1.932(7)	Li6–O5	1.92(2)
	Li6–O8	1.979(7)	Li6–O8	1.99(2)
	**Ø(Li6–O)**	**2.015(7)**	**Ø(Li6–O)**	**2.00(2)**
	Sr1–O2	2.728(2)	Sr1–O2	2.731(4)
	Sr1–O3	2.610(2)	Sr1–O3	2.633(4)
	Sr1–O4	2.564(2)	Sr1–O4	2.590(4)
	Sr1–O5	2.681(2)	Sr1–O5	2.674(4)
	Sr1–O6	2.711(2)	Sr1–O6	2.633(4)
	Sr1–O6	2.610(2)	Sr1–O6	2.679(4)
	Sr1–O8	2.688(2)	Sr1–O8	2.656(4)

Compound	$Sr[Li_3AlO_4]$		$Sr[Li_3GaO_4]$	
	Bond	Distance (Å)	Bond	Distance (Å)
	Sr1–O8	2.625(2)	Sr1–O8	2.659(4)
	**Ø(Sr1–O)**	**2.652(2)**	**Ø(Sr1–O)**	**2.657(4)**
	Sr2–O1	2.702(2)	Sr2–O1	2.703(4)
	Sr2–O1	2.532(2)	Sr2–O1	2.541(4)
	Sr2–O2	2.694(2)	Sr2–O2	2.660(4)
	Sr2–O3	2.717(2)	Sr2–O3	2.752(4)
	Sr2–O4	2.636(2)	Sr2–O4	2.644(4)
	Sr2–O5	2.628(2)	Sr2–O5	2.611(4)
	Sr2–O7	2.630(2)	Sr2–O7	2.645(4)
	Sr2–O7	2.925(3)	Sr2–O7	2.889(4)
	**Ø(Sr2–O)**	**2.683(3)**	**Ø(Sr2–O)**	**2.681(4)**

C: POWDER X-RAY DIFFRACTION

Table S7: Refined unit-cell parameters *a*, *b*, *c* (Å), *α*, *β*, *γ* (deg), and *V* (Å^3) as a function of the nominal gallium mole fraction *x* in $Sr[Li_3(Al_{1-x}Ga)O_4]$ obtained from powder X-ray diffraction data (Cu K-L_3 radiation, λ = 1.54056 Å).

x	*a* (Å)	*b* (Å)	*c* (Å)	*α* (deg)	*β* (deg)	*γ* (deg)	*V* (Å^3)
0	5.750(1)	7.329(1)	9.724(1)	83.88(1)	76.59(1)	79.57(1)	391.18(1)
0.1	5.752(1)	7.333(1)	9.727(1)	83.87(1)	76.59(1)	79.56(1)	391.62(1)
0.2	5.759(1)	7.337(1)	9.735(1)	83.90(1)	76.61(1)	79.56(1)	392.67(1)
0.4	5.776(1)	7.351(1)	9.754(1)	83.97(1)	76.66(1)	79.57(1)	395.44(1)
0.6	5.792(1)	7.364(1)	9.773(1)	84.03(1)	76.69(1)	79.58(1)	398.16(1)
0.8	5.807(1)	7.376(1)	9.791(1)	84.11(1)	76.72(1)	79.59(1)	400.66(1)
1	5.821(1)	7.387(1)	9.805(1)	84.18(1)	76.76(1)	79.59(1)	402.87(1)

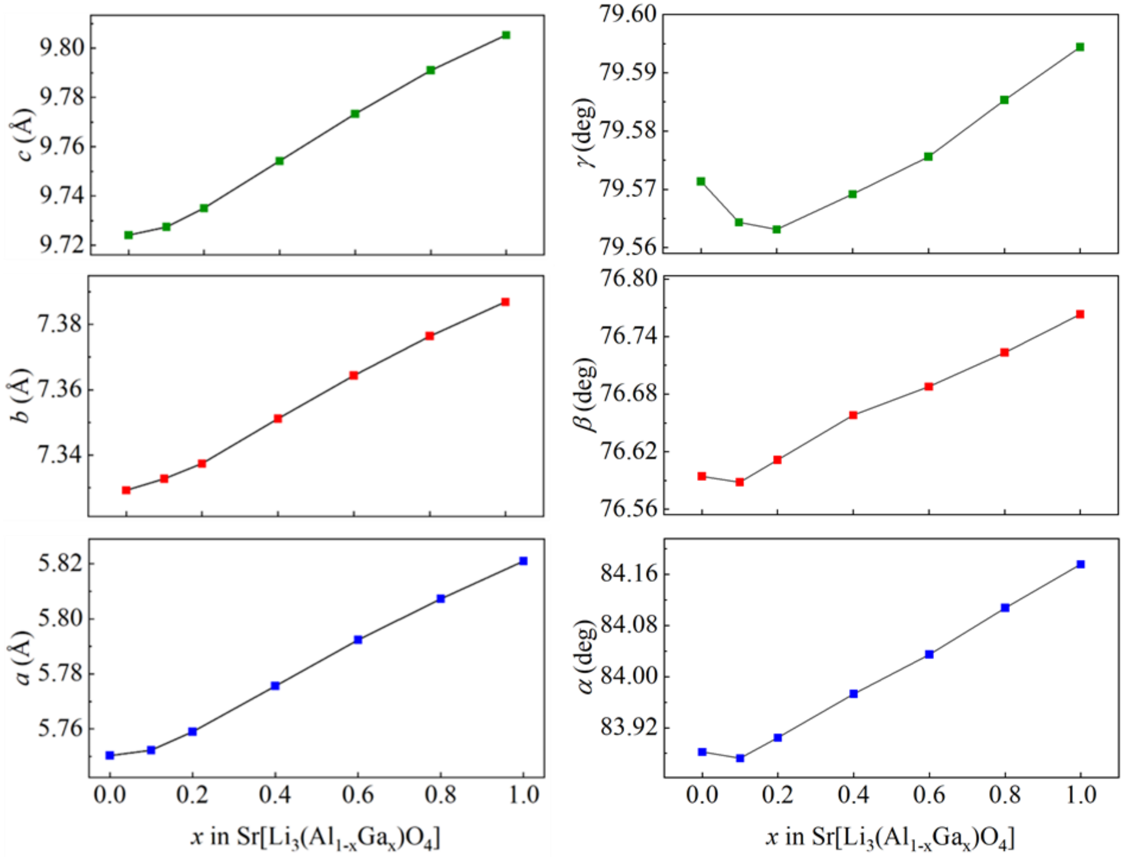

Figure S1: Graphical representations of the refined unit-cell parameters *a*, *b*, *c* (Å), *α*, *β*, and *γ* (deg) as a function of the nominal gallium mole fraction *x* in $Sr[Li_3(Al_{1-x}Ga_x)O_4]$ based on powder X-ray diffraction data (Cu K-L_3 radiation, $\lambda = 1.54056$ Å).

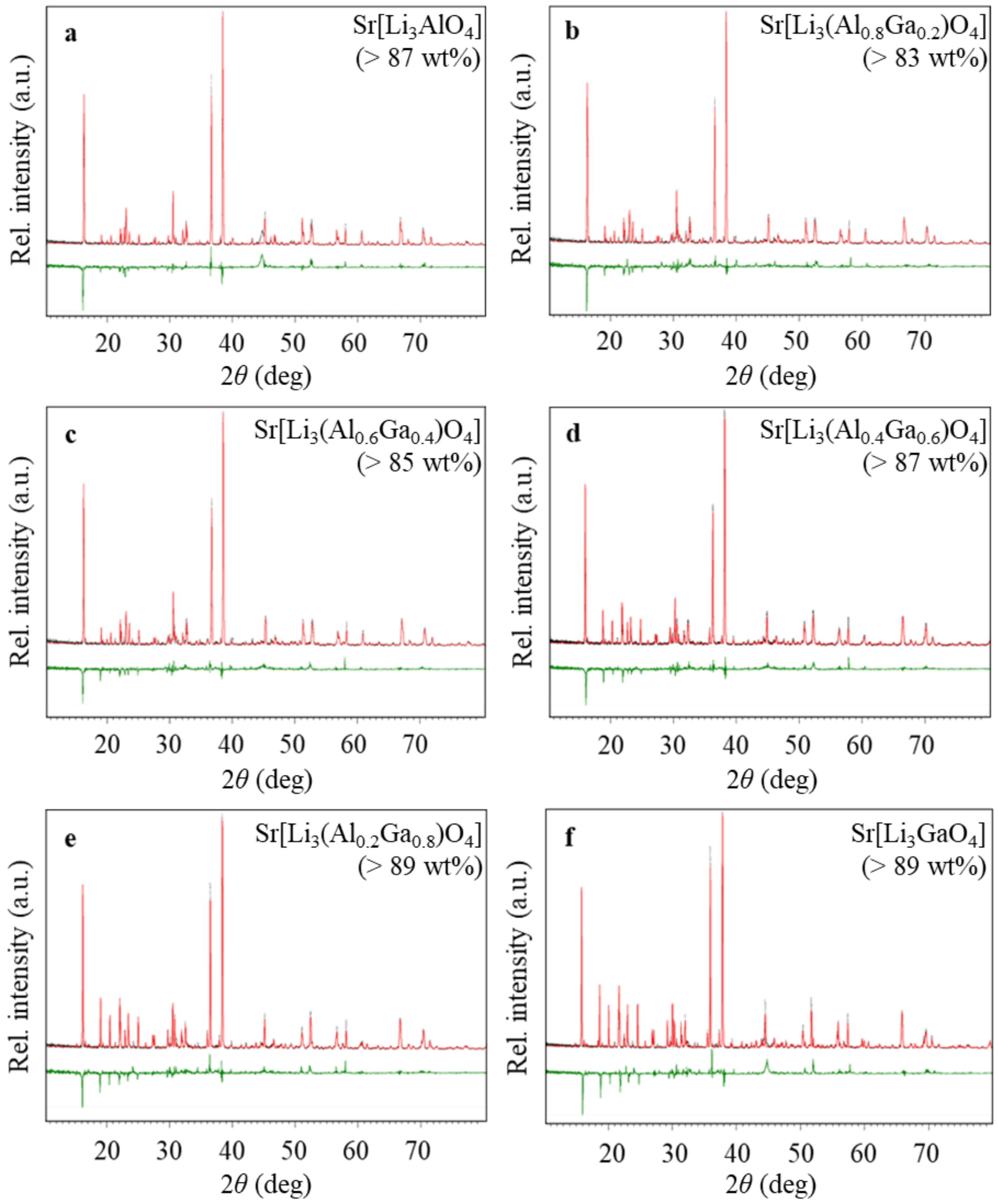

Figure S2: Rietveld refinements of powder data (Cu K-L_3 radiation, $\lambda = 1.54056$ Å) of six bulk samples of $Sr[Li_3(Al_{1-x}Ga_x)O_4]$ with the nominal gallium mole fraction x being equal to (a) 0, (b) 0.2, (c) 0.4, (d) 0.6, (e) 0.8, and (f) 1 showing the experimental X-ray diffraction data (black dots), the calculated best-fit patterns (red curves) based on the single-crystal data of $Sr[Li_3(Al/Ga)O_4]$, and the difference plots (green curves).

D: PHOTOLUMINESCENCE

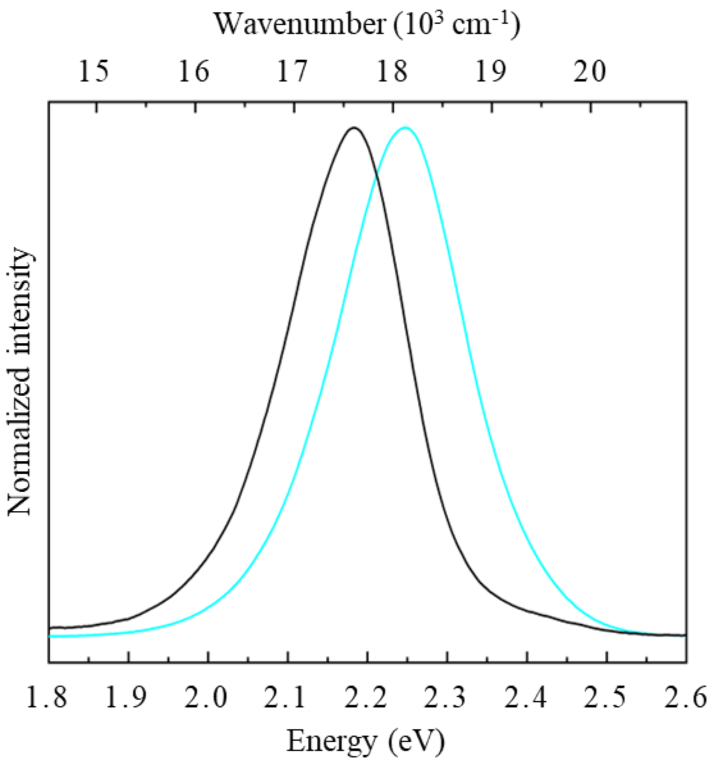

Figure S3: Normalized emission spectra of $Sr[Li_3AlO_4]:Eu^{2+}$ (black) and $Sr[Li_3GaO_4]:Eu^{2+}$ (turquoise) single crystals excited with an $In_{1-x}Ga_xN$-based laser diode (λ_{exc} = 448 nm).

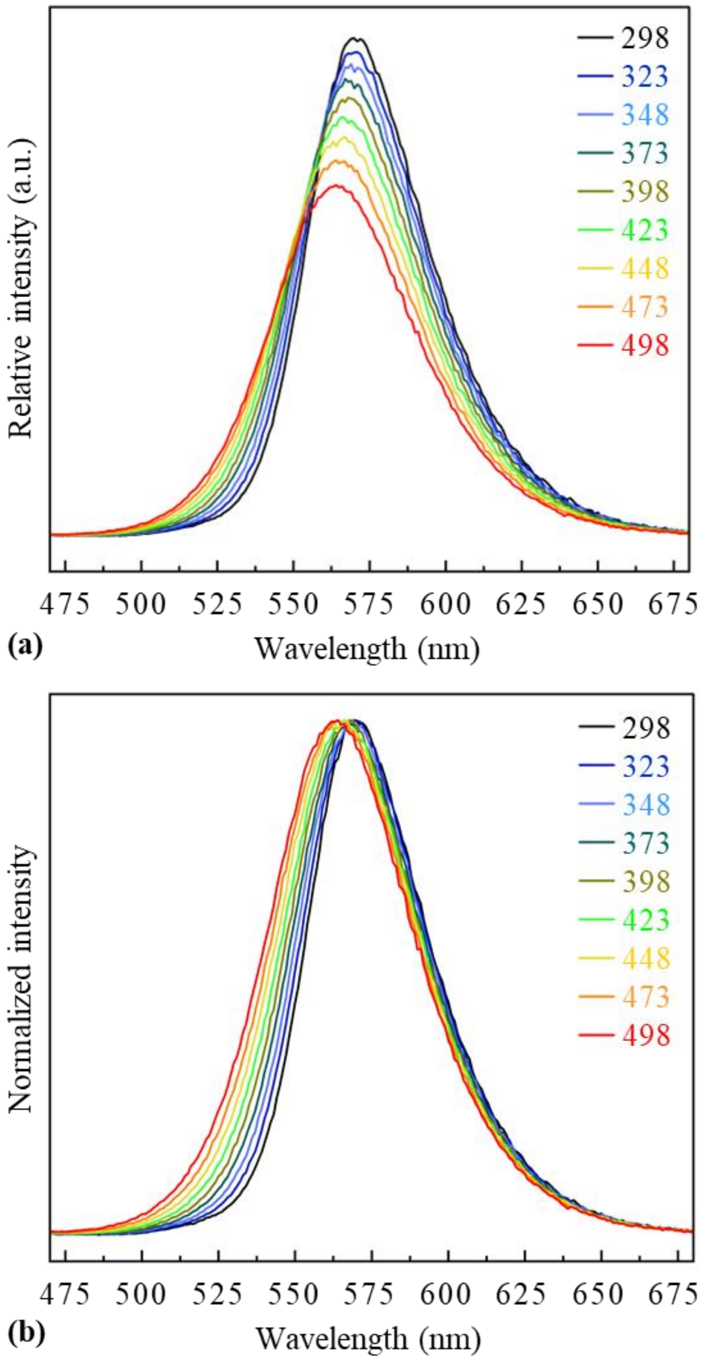

Figure S4: (a) Relative and (b) normalized photoluminescence emission spectra of $Sr[Li_3AlO_4]:Eu^{2+}$ in the temperature range from 298 to 498 K (λ_{exc} = 460 nm).

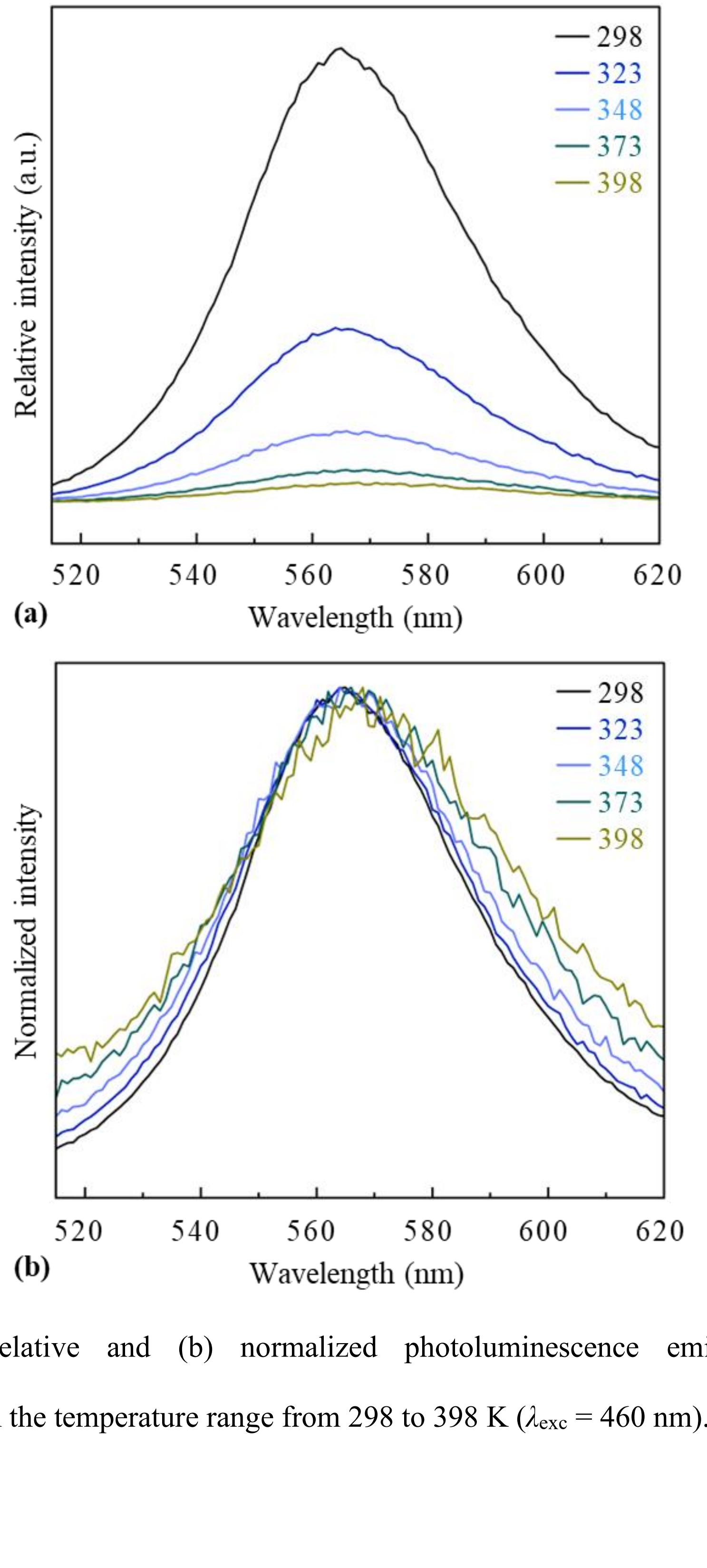

Figure S5: (a) Relative and (b) normalized photoluminescence emission spectra of $Sr[Li_3GaO_4]:Eu^{2+}$ in the temperature range from 298 to 398 K (λ_{exc} = 460 nm).

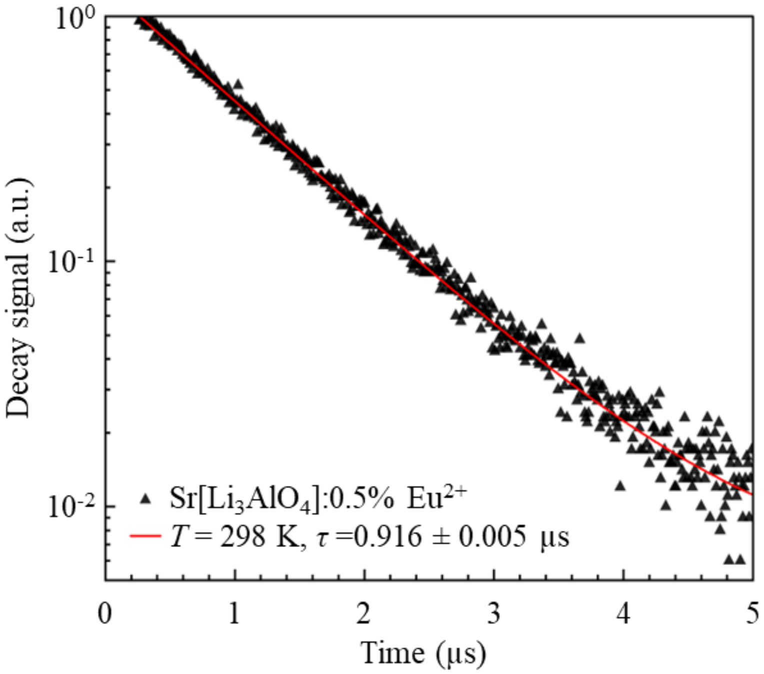

Figure S6: Semi-logarithmic plot of the luminescence decay curve of $Sr[Li_3AlO_4]:xEu^{2+}$ (x = 0.5 mol% nominal concentration) measured at room temperature upon excitation at λ_{exc} = 440 nm and emission detection at λ_{em} = 570 nm.
